# Dietary *Echinacea purpurea* polysaccharide supplementation improves antioxidative status, intestinal immune, and structure of cecal microbiota in Mahuang chickens

**DOI:** 10.3389/fvets.2026.1906481

**Published:** 2026-08-26

**Authors:** Su Peng, Zhiying Zhang, Xiaojie Huang, Yaqi Xiao, Shirui Huang, Dayou Shi

**Affiliations:** 1 College of Veterinary Medicine, South China Agricultural University, Guangzhou, China; 2 Guangxi Vocational University of Agriculture, Nanning, China

**Keywords:** antioxidant capacity, *Echinacea purpurea* polysaccharide, growth performance, immune function, intestinal health, Mahuang chicken, microbiota

## Abstract

*Echinacea purpurea* polysaccharide (EPP), a key bioactive constituent of the natural immunostimulant *E. purpurea*, shows potential as a poultry feed additive. This study evaluated EPP’s impact on growth performance, serum biochemistry indices, organ index, antioxidant capacity, immunity, intestinal health, and microbiota in Mahuang chickens. A total of 180 one-day-old Mahuang chicks (initial average body weight 39.10 ± 0.51 g) were randomly divided into six groups (six replicates, five chicks per replicate): a basal diet with 0 (CON), 0.1 g/kg levamisole hydrochloride (LMS), 100 mg/kg EPP (EPP100), 200 mg/kg EPP (EPP200), 400 mg/kg EPP (EPP400), and 800 mg/kg EPP (EPP800) for 42 days. The results showed that EPP did not affect serum biochemical indices and organ index, but significantly improved growth performance by increasing body weight gain (d 21) (*p <* 0.05). Additionally, EPP improved intestinal (duodenum, jejunum, and ileum) morphology by increasing villus height and villus height-to-crypt depth ratio, and decreasing crypt depth (d 21 and d 42) (*p <* 0.05). Com-pared to the CON and LMS groups, EPP significantly increased superoxide dismutase activity (*p =* 0.011) and total antioxidant capacity activity (*p =* 0.001) (d 21), elevated the levels of immunoglobulin (Ig) M (*p <* 0.001) and secretory IgA (*p =* 0.038) (d 21), and upregulated intestinal barrier-related genes (d 21 and d 42) (*p <* 0.05). 16S rRNA gene sequencing revealed that EPP treatment increased the Bacteroidetes/Firmicutes ratio. EPP also elevated the relative abundances of Bacteroides, Lactobacillus, Ruminococcus, Butyricicoccus, Oscillospira, and Akkermansia, while decreasing the relative abundance of Streptococcus (*p <* 0.05). Overall, the EPP groups outperformed the CON and LMS groups, with the high-dose groups (EPP400, EPP800) showing superior effects to the low-dose groups (EPP100, EPP200). In summary, this study confirmed that supplementing with EPP could boost growth performance, antioxidant capacity, immunity, intestinal barrier function, and cecal microbial community in Mahuang chickens without adverse effects, and the 400–800 mg/kg EPP appears to be optimal. This highlights the potential of EPP as a safe and effective alternative to synthetic immunostimulants in poultry production.

## 1 Introduction

In modern intensive farming systems, antibiotics were once widely used to promote poultry growth and prevent intestinal diseases (1). However, their long-term misuse has posed a serious threat to public health (2, 3). It is therefore crucial to develop safe and effective alternatives to antibiotics. Natural plant extracts have emerged as highly promising candidates for such alternatives, owing to their natural origin, low risk of inducing drug resistance, and high biosafety profile. *Echinacea purpurea* (L.) Moench (Ep) is a natural medicinal “immunostimulant” widely used in health foods, dietary supplements, and aquaculture feed (4). Existing research indicates that Echinacea powder added to the diet of one-day-old broiler chicks significantly improved the meat quality, final body weight, and enhanced their resistance to *E. coli* infection (5, 6). Furthermore, Echinacea composite ultrafine powder and Echinacea extract can increase serum immunoglobulin levels in weaned and suckling piglets (7). Other studies have shown that Echinacea extract can increase the relative abundance of probiotics in mice, improve the gut microbiota, and promote gut health (8, 9). Recent findings have demonstrated that plant polysaccharides can have multiple beneficial effects on broiler chickens: promoting growth performance, modulating immune function, enhancing antioxidant capacity, strengthening intestinal barrier integrity, and balancing the gut microbiota (10–13). *Echinacea Purpurea* polysaccharides (EPP) are one of the main active components of Echinacea and show promising potential value as a new type of green feed additive in livestock and poultry production. Additionally, levamisole, a classic synthetic immunostimulant, is widely used in poultry-related research (14–16). And it is frequently employed as a positive control for the evaluation of various immunomodulators, including both traditional Chinese medicines and Western medicines (14). Moreover, Mahuang chicken is a representative, high-quality local broiler breed in southern China, for which there is strong market demand, with annual slaughter figures reaching between 520 million and 560 million birds (17, 18). Furthermore, influenced by traditional consumption habits and rising household spending power, chilled Ma Huang chicken is more popular on the market than Arbor Acres chicken (19). Consequently, given the promising market prospects for the Mahuang chicken and the use of levamisole as a reference for evaluating immune function, investigating the application potential of EPP as a green feed additive is of considerable research value.

Preliminary studies have examined the effects of Echinacea on the growth and health of broiler chickens. However, most existing studies have focused solely on the direct administration of Echinacea powder or crude extracts, whilst the evaluation of the systemic effects of EPP at varying doses in Ma Huang chickens remains incomplete. Specifically, there remains a lack of systematic research into the combined effects of EPP on growth performance, immune function, and gut health of Mahuang chickens. Accordingly, 1-day-old Mahuang chickens were selected as experimental animals, and levamisole was set as the positive control. This study systematically investigates the effects of adding different doses of EPP (100, 200, 400, and 800 mg/kg) in the diet on growth performance, serum biochemical indices, organ indices, antioxidant capacity, immune function, intestinal morphology, mucosal barrier, and the microbiota. Furthermore, the study further evaluated the application potential of EPP as a green feed additive capable of synergistically improving animal health and provided a theoretical basis for its application in the poultry industry.

## 2 Materials and methods

### 2.1 *Echinacea purpurea* polysaccharide (EPP) preparation

The extraction of *E. purpurea* polysaccharide (EPP) by the water extraction and alcohol precipitation method referred to that described by our laboratory (20). Firstly, the *E. purpurea* was dried. After being soaked in ethanol overnight, it was then decocted with water twice. The medicinal liquids obtained from the two decoctions were combined and concentrated by rotary evaporation to make the concentration of the crude drug reach 1 g/mL. Subsequently, the impurities were removed to obtain the supernatant, which was repeatedly precipitated with alcohol three times. Finally, the polysaccharide sample of *E. purpurea* was obtained by freeze-drying and stored under refrigeration. After obtaining the EPP sample through the above extraction process, the modified phenol-sulfuric acid method was used for the determination of the sugar content (21). The polysaccharide content of EPP was calculated to be 45.76%, and the monosaccharide components of EPP are shown in Table 1 (20).

**Table 1:** The monosaccharide components of EPP.

Monosaccharide components	Content (%)
Glucose	5.79
Mannose	7.85
Arabinose	16.12
Galactose	19.84
Galacturonic acid	50.39

### 2.2 Animals, experimental design, and diets

The experiment was conducted in the South China Agricultural University to assess the effect of dietary supplementation with different levels of EPP on growth performance, intestinal histomorphology, immune status, antioxidant activity, and blood biochemical parameters of broiler chickens. All experimental procedures were approved by the Institutional Animal Care and Use Committee of South China Agricultural University (Approval No. 2025f309).

A total of 180 1-day-old ephedra Mahuang chicks were procured from the Jiangmen Kelang Agricultural Technology Co., Ltd. local hatchery. Broilers reached an average initial weight of 39.10 ± 0.51 g and were randomly allotted into six dietary treatment groups, each with 6 replicates of 5 broilers. The experiments were as follows: CON, fed with the basal diet without EPP addition; LMS, fed with the basal diet supplemented with Levamisole Hydrochloride 0.1 g/kg; EPP100, fed with the basal diet supplemented with EPP 100 mg/kg; EPP200, fed with the basal diet supplemented with EPP 200 mg/kg; EPP400, fed with the basal diet supplemented with EPP 400 mg/kg; EPP800, fed with the basal diet supplemented with EPP 800 mg/kg. The chicks were raised in an open, ventilated house with suitable litter. During the first week, the room temperature was maintained at 34 °C and gradually decreased until it reached 25 °C at the end of the rearing stage. Feed and water were provided ad libitum for 42 days (6 weeks) of the trial. A two-phase dietary regimen was implemented, the ingredients and nutrient levels of which, from day 1 to day 21 and from day 22 to day 42, are shown in Table 2. In addition, standard health and vaccination programs were conducted against Newcastle and Gumboro diseases. The chicks were observed daily, and notes were taken for any health issues.

**Table 2:** The ingredients and nutrient levels of the basal diet (air-dried basis) %.

Component	Days 1–21	Days 22–42
Ingredient (%)		
Corn	54.50	55.30
Soybean meal (CP, 43%)	33.00	25.50
Fish meal	1.00	0.00
Wheat bran	5.50	5.00
Soy oil	1.50	4.00
Rapeseed meal	0.00	6.00
Limestone powder	1.40	1.30
CаHPO_4_	1.10	0.90
Premix^1^	2.00	2.00
Total	100.00	100.00
Nutrient levels (%)^2^		
Crude protein	21.00	19.50
Calcium	1.05	0.90
Total phosphorus	0.50	0.43
Lysine	1.20	1.12
Methionine	0.55	0.50
Threonine	0.19	0.18
Metabolizable energy, kcal/kg	2920.58	3020.96

^1^The premix provided the following per kilogram of diet: VA, 7500 IU; VB_1_, 3.5 mg; VB_2_, 3.2 mg; VB_5_, 22 mg; VB_6_, 3.2 mg; VB_12_, 0.02 mg; VD_3_, 1,500 IU; VK_3_, 2 mg; biotin, 0.12 mg; folic acid, 1.0 mg; pantothenic acid, 28.0 mg; nicotinic acid, 18 mg; antioxidant, 100 mg; Cu, 6.5 mg; Fe, 70 mg; Mn, 60 mg; Zn, 60 mg; Se, 0.4 mg. ^2^Metabolizable energy was a calculated value, while the others were measured values.

### 2.3 Growth performance

Chickens were weighed individually on the first day of age to determine the average initial body weight (IBW) and then weighed at 7, 14, 21, 28, 35, and 42 days of age to calculate the average body weight of the chickens in each group. At each interval, the body weight gain (BWG) was calculated by subtracting the IBW from the final body weight (FBW) within the same period.

The BWG, feed intake (FI), and feed conversion ratio (FCR) were calculated as per the following equations:

BWG (g) = FBW(g) − IBW (g)


$$\mathrm{FI}(g)=\frac{\text{The amount of feed offered}-\text{feed residues}}{\mathrm{No}.\text{of chickens in each replicate}}$$

$$\mathrm{FCR}=\frac{\mathrm{FI}(g)}{\mathrm{BWG}(g)}$$

### 2.4 Sample collection

On days 21 and 42 of the trial, one bird was randomly selected from each replicate and euthanized by cervical dislocation, according to the American Veterinary Medical Association guidelines (22). Then, blood samples were collected into sterilized tubes without anticoagulant and left to clot at room temperature. The samples were centrifuged for 15 min at 3,500 rpm (4 °C) to separate the serum and were stored at −80 °C for biochemical indices, antioxidant capacity, and immunoglobulin analysis. Samples from the middle of the duodenum, jejunum, and ileum (1 cm) were harvested and fixed in 10% formalin for histomorphological examination. Full-thickness duodenum, jejunum, and ileum resected and washed with cold PBS were opened longitudinally, collected, frozen immediately in liquid nitrogen, and then stored at −80 °C to determine antioxidants and immunoglobulin, as well as for the mRNA expression analysis. The cecal contents from 42-day-old chickens (*n* = 4 per group) were quickly collected into 2 mL cryovials, flash-frozen in liquid nitrogen, and rapidly stored at −80 °C for subsequent microbiological analysis.

### 2.5 Serum biochemical indices

The serum biochemical indices were determined using the serum samples collected on days 21 and 42 of the experiment. Employing an automatic biochemical analyzer, the levels of UREA, Alkaline Phosphatase (ALP), Aspartate Aminotransferase (AST), Alanine Aminotransferase (ALT), Total Cholesterol (TC), Triglyceride (TG), Albumin (ALB), Total Protein (TP), and Globulin (GLB) in the serum of ephedra broilers were measured.

### 2.6 Organ index

After blood collection, the chickens were dissected. The spleen, bursa of Fabricius, and thymus were carefully removed. Thereafter, these organs were cleaned and weighed to calculate the organ indices. Organ index (g/kg) = spleen, thymus, or bursa weight (g)/body weight (kg).

### 2.7 Antioxidant capacity

Antioxidant parameters, such as total superoxide dismutase (T-SOD), total antioxidant capacity (T-AOC), and malondialdehyde (MDA) in the serum, duodenum, jejunum, and ileum, were tested. Serum samples should be used after centrifugation for 15 min at 3,500 rpm (4 °C). Add 9 times the volume of saline solution to the intestinal sample in a ratio of weight (g): volume (mL) = 1: 9. Prepare a 10% homogenate in an ice-water bath, then centrifuge at 4,000 rpm for 10 min and collect the supernatant for analysis. All analyses were carried out in duplicate using kits from Nanjing Jiancheng Bioengineering Institute (Nanjing, China). Every step strictly adhered to the manufacturer’s instructions.

### 2.8 Immunoglobulin

Enzyme-linked immunosorbent assay (ELISA) using kits from Shanghai Enzyme-linked Biotechnology Co., Ltd. (Shanghai, China) was employed to determine the contents of immunoglobulins (IgA, IgG, and IgM) in samples from serum, duodenum, jejunum, and ileum. Serum samples are allowed to clot for 30 min at room temperature, then centrifuged for 15 min at 3,500 rpm (4 °C) before use. The intestinal tissue was rinsed thoroughly with pre-chilled PBS. Nine times the volume of PBS was added at a ratio of weight (g): volume (mL) = 1: 9. A 10% homogenate was prepared in an ice-water bath and centrifuged at 5,000 × g for 10 min. Collect the supernatant for analysis. All testing procedures were performed strictly in accordance with the manufacturer’s official instructions.

### 2.9 Intestinal morphology

Two-centimeter samples (six samples /group) of the small intestine (duodenum, jejunum, and ileum) had been fixed in 10% formalin for histomorphological examination. Briefly, they were dehydrated for 72 h. Then clearing and embedded in paraffin. Transverse sections were fixed on slides, then stained with hematoxylin(H&E) and eosin, and sealed. Intestinal villus height (VH) was measured (μm) from the tip (with a lamina propria) to the base of the villus (villus-crypt junction), and the crypt depth (CD) was calculated from the villus-crypt junction to the distal limit of the crypt. They were measured using the digital slice scanning software NDP. view 2. Finally, the average value and the ratio of VH to CD were calculated to assess the digestive and absorptive capacity of the intestine (23).

### 2.10 Real-time quantitative polymerase chain reaction (RT qPCR) analysis

Total RNA was extracted from the samples using the silica gel column purification technology of the RNApure Fast Tissue & Cell Kit (Cat. No. CW0599S, CWBIO, Jiangsu, China). The concentration and purity of the extracted RNA were determined with a micro-ultraviolet–visible spectrophotometer (NanoDrop-2000). Subsequently, 1 μg of total RNA was used to synthesize cDNA with HiFi Script gDNA Removal RT Master Mix (CW2020M, CWBIO, Jiangsu, China). Real-time qPCR was performed using SuperStar Universal SYBR Master Mix (Cat. No. CW3360, CWBIO, Jiangsu, China) following the manufacturer’s instructions. The specific fluorescence quantitative PCR reaction system is indicated in Table 3. The RT-qPCR cycling conditions are indicated in Table 4. The primers for tight junction protein and immune-related genes were synthesized by Sangon Biotech Co., Ltd. (Shanghai, China). The specific primer sequences of the target genes are presented in Table 5. The relative expression of the target gene for each sample was analyzed through the formula 2^−(∆∆Ct)^, where ∆∆Ct = (Ct_target_ − Ct_β-actin_) _treatment_ − (Ct_target_ − Ct_β-actin_) _control_ (23).

**Table 3:** The configuration of the reaction liquid.

Reagents	Added amount
2 × SuperStar Universal SYBR Master Mix	10 μL
Forward primer, 10 μM	0.4 μL
Reverse primer, 10 μM	0.4 μL
Template cDNA	2.0 μL
Double-distilled water	7.2 μL

**Table 4:** qPCR amplification parameters.

Step	Temperature	Time
Pre-transformation	95 °C	30 s
Transsexual	95 °C	5 s × 40 cycles
Annealing/extension	60 °C	30 s* × 40 cycles
	95 °C	15 s
Melting curve	60 °C	1 min*
	95 °C	1 s

**Table 5:** Target genes and primer sequences used for quantification in this study.

Genes	Forward primer sequence 5′–3′	Reverse primer sequence 5′–3′	Accession number
β-actin	GAGAAATTGTGCGTGACATCA	CCTGAACCTCTCATTGCCA	NM_205518
Occludin	TCATCCTGCTCTGCCTCATCT	CATCCGCCACGTTCTTCAC	NM_205128.1
Claudin1	GAGGATGACCAGGTCAAGAAG	TGCCCAGCCAATGAAGAG	NM_001013611.2
Claudin2	CACCGTCTTCAATCAGGGCT	AGCTGAACTCACTCTTGGGC	NM_001277622.1
ZO-1	CCAAAGACAGCAGGAGGAGA	TGGCTAGTTTCTCTCGTGCA	XM_015278981.1
MUC2	CATTCAACGAGGAGAGCTGC	TTCCTTGCAGCAGGAACAAC	NM_001318434.1
TLR4	AGTCTGAAATTGCTGAGCTCAAAT	GCGACGTTAAGCCATGGAAG	NM_001030693.1
MyD88	AGCATTACCAGGGCTGAGTT	TGGTACCATGCCAGCAGTTA	NM_001030962.4
TNF-α	AATTTGCAGGCTGTTTCTGC	TATGAAGGTGGTGCAGATGG	NM_204267.1
IFN-γ	GGACATGGCTCCCACACTAC	TGAAGAGGTGCTGAAGGATG	NM_205427.1
IL-1β	GAAGTGCTTCGTGCTGGAGT	ACTGGCATCTGCCCAGTTC	NM_204524.1
NF-κB-p65	CAGCCCATCTATGACAACCG	TCAGCCCAGAAACGAACCTC	NM_001396038.1
Nrf2	ATCACCTCTTCTGCACCGAA	GCTTTCTCCCGCTCTTTCTG	NM_205117.1
iNOS	CCTGTACTGAAGGTGGCTATTGG	AGGCCTGTGAGAGTGTGCAA	D85422

*β*-actin = The internal reference gene beta actin; ZO-1 = Zonula occludens-1; MUC2 = Mucin 2; TLR4 = Toll-like receptor 4; MyD88 = myeloid differentiation factor 88; TNF-α = tumor necrosis factor-α; IFN-γ = Interferon-gamma; IL-1β = interleukin-1β; NFκB-P65 = nuclear factor-kappa B-P65; Nrf2 = Nuclear factor erythroid 2-related factor 2; iNOS = Inducible nitric oxide synthase.

### 2.11 Gut microbiota profiling and analysis

Total microbial DNA was extracted from the cecal contents of four randomly selected broilers from each group at 42 days of age using the MagBeads FastDNA Kit (MP Biomedicals, CA, USA). Each chicken was pre-assigned a unique number, and a random number table method was adopted to complete random selection without artificial preference. PCR was used to amplify the bacterial 16S rRNA gene’s V3-V4 region (forward primer: 5′-ACTCCTACGGGAGGCAGCA-3′ and reverse primer: 5′-GGACTACHVGGGTWTCTAAT-3′). PCR products were purified using Vazyme VAHTST’M DNA Clean Beads (Vazyme, Nanjing, China), and quantification was performed using a QuantiT PicoGreen dsDNA Assay Kit (Invitrogen, Carlsbad, CA, USA). 16 s rRNA sequencing was performed using the Illumina Novaseq_PE250 (Illumina, San Diego, CA) sequencing platform; sequencing services were provided by Personal Biotechnology Co., Ltd. (Shanghai, China). Data collection and microbial analysis were completed using the free online platform Genescloud,^1^ including operational taxonomic unit (OTU) clustering, principal coordinate analysis (PCoA), non-metric multidimensional scaling (NMDS), species annotation, and linear discriminant analysis effect size (LefSe), ultimately obtaining information on microbial community composition and differential species.

### 2.12 Statistical analysis

We first sorted all experimental data with Excel 2019 software. The data were conducted one-way analysis of variance (one-way ANOVA) with the ANOVA module in IBM SPSS Statistics 27.0 software (IBM Corp., Armonk, NY, USA). GraphPad Prism 8.0 software (GraphPad, San Diego, CA) was employed to generate graphs. Duncan’s multiple range test was applied to perform multiple comparisons among groups. The results in the tables were expressed as mean and pooled mean standard error (SEM). A *p*-value < 0.05 indicates a significant difference.

## 3 Results

### 3.1 Growth performance

**Figure 1:**
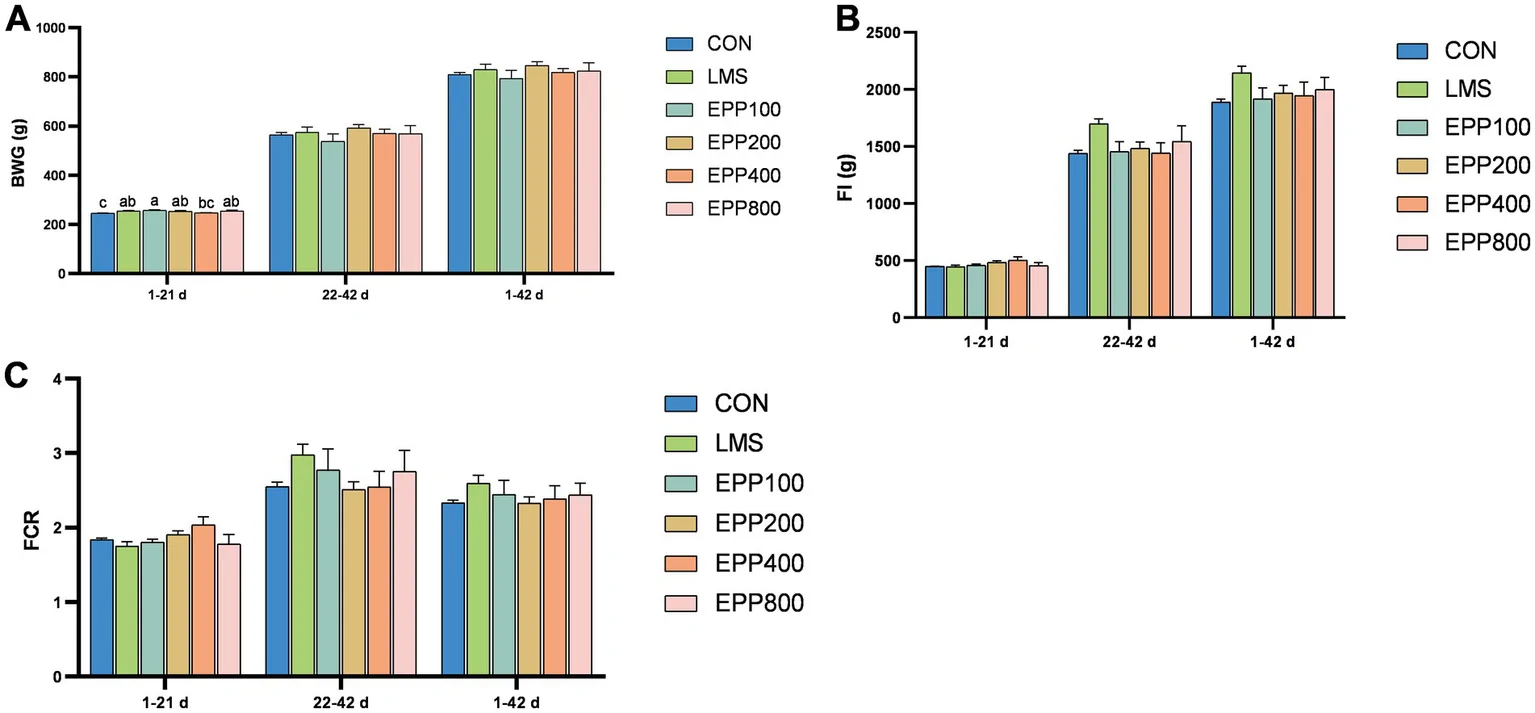
Effects of dietary Echinacea purpurea polysaccharide (EPP) on growth performance in Mahuang chickens. **(A)** Body weight gain, **(B)** feed intake, and **(C)** feed conversion ratio. CON, fed with the basal diet; LMS, basal diet + Levamisole Hydrochloride 0.1 g/kg; EPP100, the basal diet + EPP 100 mg/kg; EPP200, the basal diet + EPP 200 mg/kg; EPP400, the basal diet + EPP 400 mg/kg; EPP800, the basal diet + EPP 800 mg/kg. Bars represent mean values ± SEM. The regressions were considered significant at *p*-value < 0.05. ^a,b,c^Means within the same row carrying different superscripts were significantly different at p-value < 0.05, while identical superscripts or no superscripts indicate no significant difference at *p*-value >0.05.

During the experiment, the broiler house was cleaned regularly, and no adverse events occurred. Figure 1 presents the effects of dietary *E. purpurea* polysaccharide (EPP) on the growth performance of Mahuang chickens. From 1 to 21 days, the BWG in the LMS, EPP100, EPP200, and EPP800 groups was significantly higher than that in the CON group (*p =* 0.011). However, there were no significant differences in BWG, FI, and FCR across all groups at 22–42 days and 1–42 days (*p >* 0.05). In summary, supplementation with EPP significantly improved BWG of broilers during the 1–21 d, whereas no obvious differences were observed in overall FI and FCR throughout the whole experimental period.

### 3.2 Organ index

The effects of diets with different levels of EPP on the organ indices of spleen, bursa, and thymus in broiler chickens are shown in Table 6. Overall, no significant differences were detected in the organ indices of these Mahuang broilers at both 21 and 42 days old (*p >* 0.05). This indicates that, under the conditions of this experiment, no dose-dependent changes in the development of immune organs were observed across groups.

**Table 6:** Effects of dietary EPP on organ index in Mahuang chickens (g/kg).

Item	CON	LMS	EPP100	EPP200	EPP400	EPP800	SEM	*p*-value
21 days old								
Spleen index	1.33	1.46	1.40	1.20	1.66	1.19	0.056	0.129
Bursa index	4.03	3.56	3.71	3.39	4.11	3.72	0.189	0.890
Thymus index	5.51	5.46	5.46	5.22	5.86	6.34	0.296	0.923
42 days old								
Spleen index	1.65	1.86	1.35	1.04	1.44	1.32	0.137	0.650
Bursa index	2.98	3.55	2.50	2.19	3.11	3.20	0.209	0.474
Thymus index	4.91	4.70	4.20	4.11	4.95	4.26	0.218	0.809

CON = fed with the basal diet; LMS = basal diet + Levamisole Hydrochloride 0.1 g/kg; EPP100 = the basal diet + EPP 100 mg/kg; EPP200 = the basal diet + EPP 200 mg/kg; EPP400 = the basal diet + EPP 400 mg/kg; EPP800 = the basal diet + EPP 800 mg/kg. Variation in the data was expressed as pooled SEM. The regressions were considered significant at *p*-value < 0.05.

### 3.3 Serum biochemical indices

The effects of dietary EPP on the serum biochemical indices of broiler chickens are shown in Table 7. There were no significant differences in the serum biochemical levels of UREA, ALP, AST, ALT, TC, TG, ALB, TP, and GLB among the experimental groups at 21 and 42 days of age (*p >* 0.05). This suggests that EPP had no adverse impact on systemic hepatic and renal metabolic homeostasis of Mahuang broilers.

**Table 7:** Effects of dietary EPP on serum biochemical indices in Mahuang chickens.

Item	CON	LMS	EPP100	EPP200	EPP400	EPP800	SEM	*p*-value
21 days old								
UREA, mmol/L	0.64	0.63	0.53	0.53	0.49	0.53	0.022	0.231
ALP, U/L	1556.33	2249.55	1935.51	1878.55	2921.04	4139.10	286.831	0.124
AST, U/L	248.86	262.94	260.96	237.14	250.51	255.45	6.132	0.869
ALT, U/L	5.44	5.35	6.13	5.25	5.93	6.80	0.159	0.055
TC, mg/dL	3.66	3.72	3.85	3.86	3.90	4.11	0.094	0.816
TG, mmol/L	0.31	0.31	0.32	0.34	0.29	0.35	0.013	0.788
ALB, g/dL	11.28	12.05	11.26	10.43	10.24	10.73	0.269	0.409
TP, g/dL	29.56	35.20	30.73	28.84	29.24	30.48	0.959	0.439
GLB, g/dL	18.38	23.25	19.38	18.50	19.00	19.75	0.737	0.425
42 days old								
UREA, mmol/L	0.52	0.53	0.53	0.53	0.49	0.56	0.026	0.987
ALP, U/L	3727.43	2868.15	3237.12	4095.40	2346.38	3263.63	290.329	0.611
AST, U/L	229.08	241.35	238.87	254.37	231.32	241.60	4.116	0.579
ALT, U/L	5.82	7.28	7.05	7.28	7.08	7.15	0.177	0.129
TC, mg/dL	4.04	4.21	3.87	4.20	4.32	4.44	0.079	0.373
TG, mmol/L	0.29	0.27	0.31	0.36	0.34	0.28	0.013	0.253
ALB, g/dL	11.03	10.65	10.77	10.87	10.65	10.82	0.160	0.987
TP, g/dL	29.47	29.78	27.70	31.57	27.88	29.48	0.610	0.507
GLB, g/dL	18.33	19.17	17.00	20.67	17.33	18.67	0.502	0.333

CON = fed with the basal diet; LMS = basal diet + Levamisole Hydrochloride 0.1 g/kg; EPP100 = the basal diet + EPP 100 mg/kg; EPP200 = the basal diet + EPP 200 mg/kg; EPP400 = the basal diet + EPP 400 mg/kg; EPP800 = the basal diet + EPP 800 mg/kg. ALP, alkaline phosphatase; AST, aspartate aminotransferase; ALT, alanine aminotransferase; TC, total cholesterol; TG, triglyceride; ALB, albumin; TP, total protein; GLB, globulin. Variation in the data was expressed as pooled SEM. The regressions were considered significant at *p*-value < 0.05.

### 3.4 Serum antioxidant capacity

**Figure 2:**
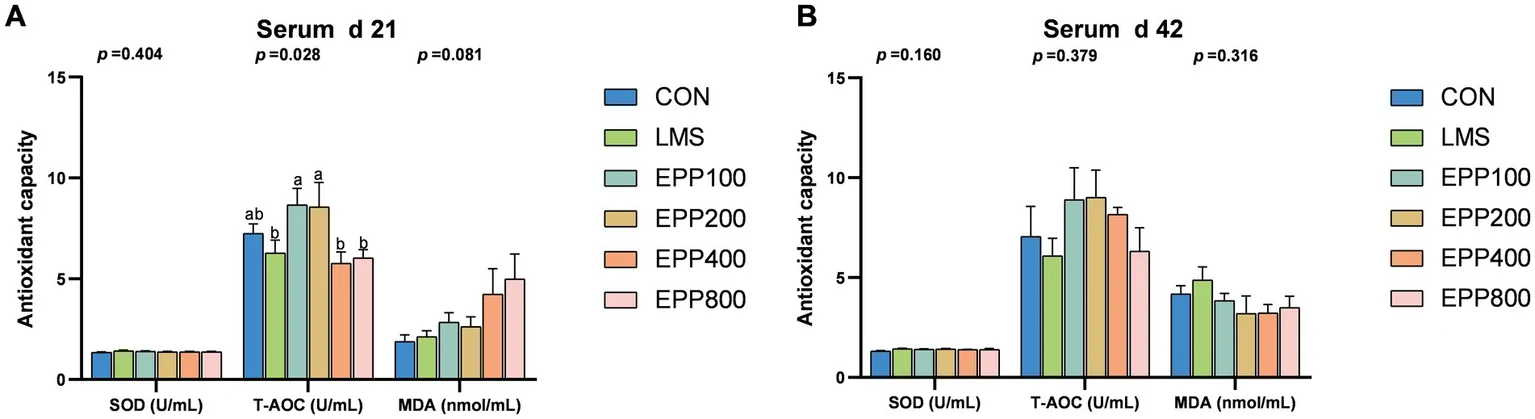
Effects of EPP on serum antioxidant capacity of Mahuang chickens. **(A,B)** The antioxidant capacity indices (SOD, superoxide dismutase; T-AOC, total antioxidant capacity; and MDA, malondialdehyde) in the serum, duodenum, jejunum, and ileum at days 21 **(A)** and 42 **(B)**. CON, fed with the basal diet; LMS, basal diet + Levamisole Hydrochloride 0.1 g/kg; EPP100, the basal diet + EPP 100 mg/kg; EPP200, the basal diet + EPP 200 mg/kg; EPP400, the basal diet + EPP 400 mg/kg; EPP800, the basal diet + EPP 800 mg/kg. Bars represent mean values ± SEM. The regressions were considered significant at *p*-value < 0.05. ^a,b^Means within the same row carrying different superscripts were significantly different at *p*-value < 0.05, while identical superscripts or no superscripts indicate no significant difference at *p*-value >0.05.

The impact of EPP on the activities of serum antioxidant capacity of Mahuang chickens is shown in Figure 2. Compared with the CON group, there were no significant differences in serum antioxidant indices among the groups at 21 days (Figure 2A) and 42 days (Figure 2B) (*p >* 0.05). Compared with the LMS group, the 21-day serum T-AOC activity for the EPP100 and EPP 200 groups was significantly higher (*p =* 0.028). The results indicate that the addition of EPP to the diet on the antioxidant capacity was superior to, or equivalent to, that of LMS in MaHuang chickens.

### 3.5 Serum immunoglobulin

**Figure 3:**
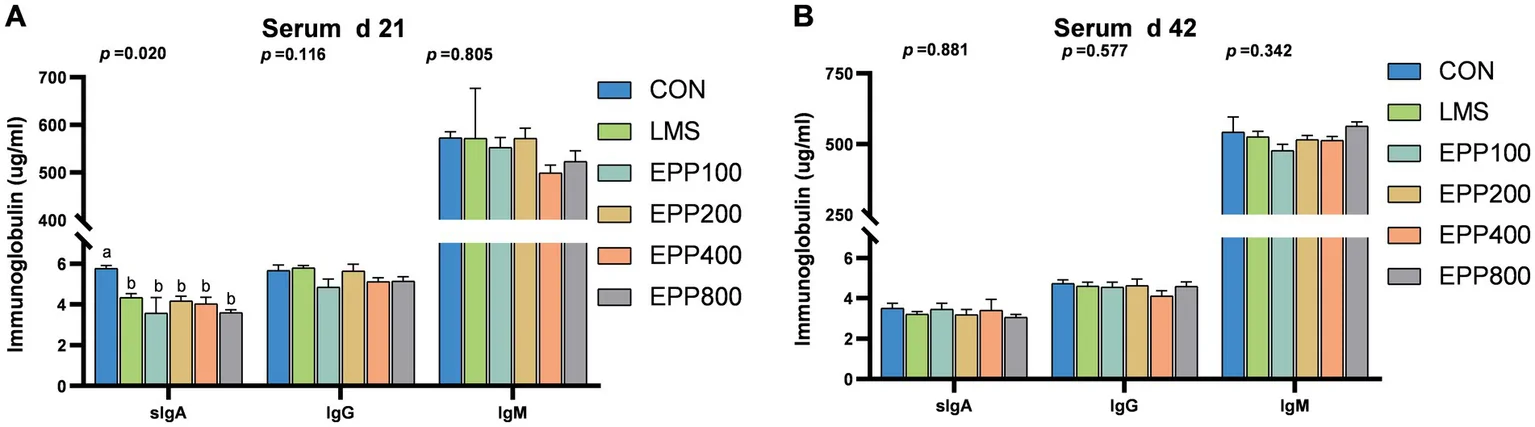
Effects of dietary EPP on serum immunoglobulin in Mahuang chickens. **(A,B)** The immunoglobulin indices (sIgA, Secretory Immunoglobulin A; IgG, Immunoglobulin G; IgM, Immunoglobulin M) in the serum at days 21 **(A)** and 42 **(B)**. CON, fed with the basal diet; LMS, basal diet + Levamisole Hydrochloride 0.1 g/kg; EPP100, the basal diet + EPP 100 mg/kg; EPP200, the basal diet + EPP 200 mg/kg; EPP400, the basal diet + EPP 400 mg/kg; EPP800, the basal diet + EPP 800 mg/kg. Bars represent mean values ± SEM. The regressions were considered significant at *p*-value < 0.05. ^a,b^Means within the same row carrying different superscripts were significantly different at *p*-value < 0.05, while identical superscripts or no superscripts indicate no significant difference at *p*-value >0.05.

The effects of dietary EPP on the levels of serum immunoglobulins in the Mahuang chickens are shown in Figure 3. At 21 days of age, serum sIgA levels in all treatment groups were significantly lower than those in the CON group (*p =* 0.020) (Figure 3A). But there were no significant differences in IgG and IgM levels among the groups (*p >* 0.05). At 42 days of age, there were no significant differences in serum immunoglobulin levels among the groups (*p >* 0.05) (Figure 3B). This indicates that EPP and LMS have consistent effects on serum immunoglobulins.

### 3.6 Intestinal morphology

**Figure 4:**
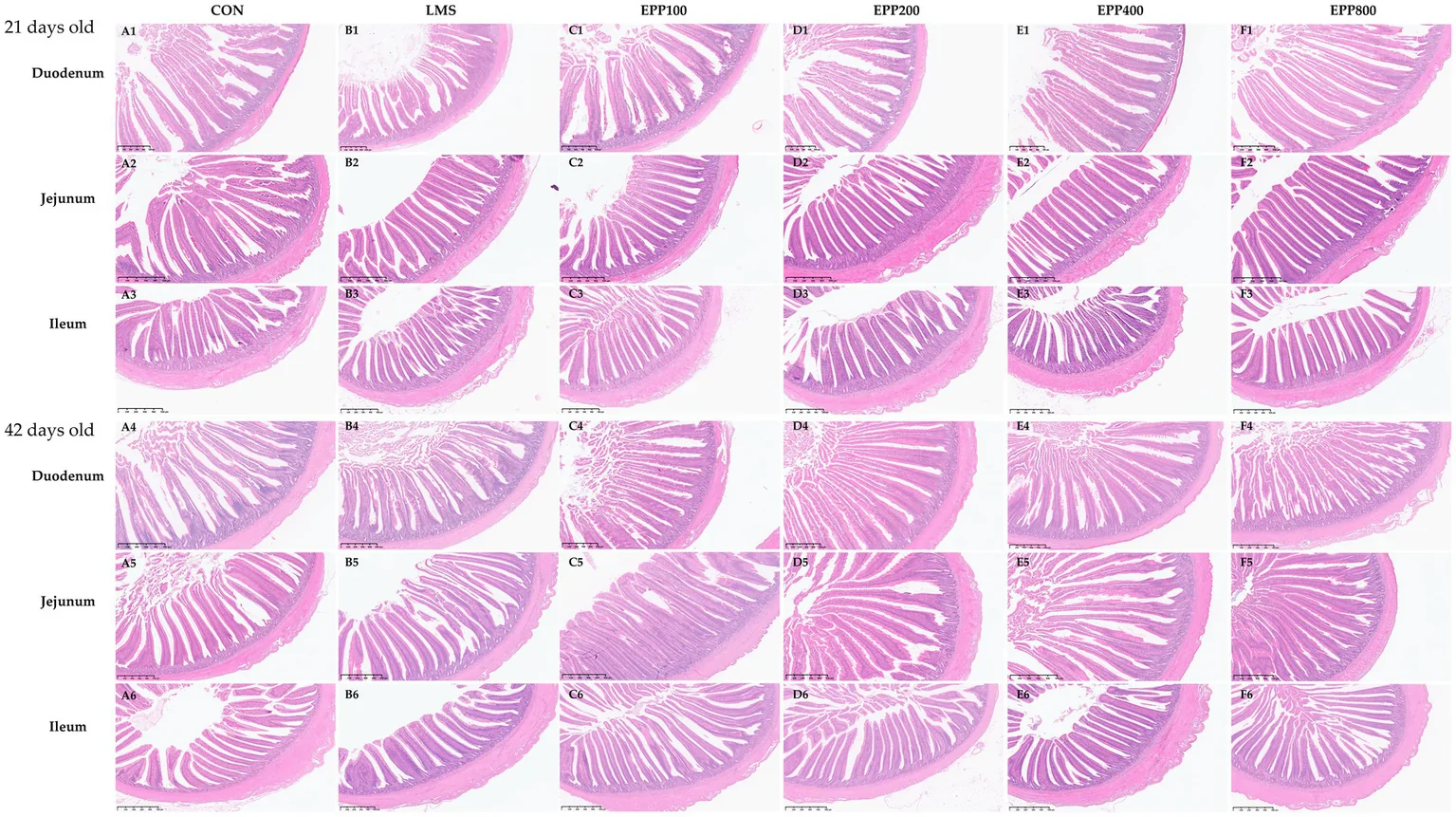
The small intestinal morphology in Mahuang chickens. CON, fed with the basal diet; LMS, basal diet + Levamisole Hydrochloride 0.1 g/kg; EPP100, the basal diet + EPP 100 mg/kg; EPP200, the basal diet + EPP 200 mg/kg; EPP400, the basal diet + EPP 400 mg/kg; EPP800, the basal diet + EPP 800 mg/kg. Photomicrograph of the small intestine: 21 days old duodenum **(A1–F1)**, jejunum **(A2–F2)**, and ilium **(A3–F3)**; 42 days old duodenum **(A4–F4)**, jejunum **(A5–F5)**, and ilium **(A6–F6)** stained with hematoxylin and eosin stain; CON **(A1–A6)**, LMS **(B1–B6)**, EPP100 **(C1–C6)**, EPP200 **(D1–D6)**, EPP400 **(E1–E6)**, EPP800 **(F1–F6)**.

Table 8 and Figure 4 depict the results of intestinal morphometric measurements in Mahuang chickens. Throughout the experimental period (days 21 and 42), dietary supplementation with EPP had an effect on the VH, CD, and VH/CD in small intestinal morphology (Table 8). On day 21, the duodenal VH (*p <* 0.001) and VH/CD (*p <* 0.001) in the EPP200, EPP400, and EPP800 groups increased more remarkably than in the CON group. The CD in the EPP group was significantly higher than in the CON and LMS groups. In the jejunum, the EPP800 group demonstrated a substantial increase in VH compared to other groups (*p <* 0.001). Moreover, the VH/CD in the LMS, EPP100, EPP400, and EPP800 groups was significantly higher than that in the CON group (*p =* 0.019). In the ileum, compared with the CON and LMS groups, the EPP group showed a significant increase in VH and VH/CD and a significant decrease in CD (*p <* 0.001).

**Table 8:** Effects of dietary EPP on small intestinal morphology in Mahuang chickens.

Item	CON	LMS	EPP100	EPP200	EPP400	EPP800	SEM	*p*-value
21 days old								
Duodenum								
VH, μm	1155.41^c^	1205.93^bc^	1200.93^bc^	1287.41^a^	1274.89^ab^	1296.00^a^	10.383	<0.001
CD, μm	146.51^a^	131.83^b^	111.93^c^	107.74^c^	107.99^c^	104.31^c^	1.239	<0.001
VH/CD	9.64^d^	10.97^cd^	10.28^cd^	11.84^bc^	13.16^ab^	14.50^a^	0.363	<0.001
Jejunum								
VH, μm	879.83^bc^	886.53^bc^	836.05^c^	834.27^c^	895.49^b^	1060.82^a^	7.792	<0.001
CD, μm	145.71^a^	107.83^b^	108.08^b^	114.20^b^	106.67^b^	97.95^c^	1.180	<0.001
VH/CD	6.03^b^	8.45^a^	7.91^a^	7.30^ab^	8.34^a^	9.19^a^	0.286	0.019
Ileum								
VH, μm	667.72^b^	674.43^b^	748.66^a^	756.94^a^	753.97^a^	761.86^a^	4.655	<0.001
CD, μm	159.38^a^	139.93^b^	114.78^c^	111.89^cd^	115.34^c^	105.00^d^	1.227	<0.001
VH/CD	4.20^b^	4.97^b^	6.76^a^	6.66^a^	6.98^a^	7.44^a^	0.277	<0.001
42 days old								
Duodenum								
VH, μm	1439.67^ab^	1441.85^ab^	1409.19^b^	1501.17^a^	1473.73^ab^	1522.83^a^	10.649	0.038
CD, μm	168.01^a^	172.39^a^	129.95^b^	124.44^b^	124.70^b^	123.81^b^	1.519	<0.001
VH/CD	7.02^b^	7.52^b^	10.54^a^	12.15^a^	10.89^a^	10.89^a^	0.405	<0.001
Jejunum								
VH, μm	1135.84^c^	1116.00^c^	1334.49^a^	1240.04^b^	1302.74^ab^	1309.32^ab^	11.912	<0.001
CD, μm	156.55^a^	146.60^a^	105.49^b^	99.39^b^	110.70^b^	103.40^b^	2.269	<0.001
VH/CD	7.37^b^	8.04^b^	13.15^a^	12.86^a^	12.12^a^	13.41^a^	0.566	<0.001
Ileum								
VH, μm	767.22^c^	801.90^c^	939.34^a^	893.90^ab^	890.45^b^	862.38^b^	6.719	<0.001
CD, μm	152.40^a^	141.96^b^	105.96^cd^	102.67^d^	112.46^c^	92.91^e^	1.248	<0.001
VH/CD	5.19^b^	5.86^b^	8.95^a^	8.62^a^	7.97^a^	9.28^a^	0.334	<0.001

CON = fed with the basal diet; LMS = basal diet + Levamisole Hydrochloride 0.1 g/kg; EPP100 = the basal diet + EPP 100 mg/kg; EPP200 = the basal diet + EPP 200 mg/kg; EPP400 = the basal diet + EPP 400 mg/kg; EPP800 = the basal diet + EPP 800 mg/kg. VH, villus height; CD, crypt depth; VH/CD, villus height to crypt depth. Variation in the data was expressed as pooled SEM. The regressions were considered significant at *p*-value < 0.05. ^a,b,c,d^Means within the same row carrying different superscripts were significantly different at *p*-value < 0.05, while identical superscripts or no superscripts indicate no significant difference at *p*-value >0.05.

By day 42, compared to the CON and LMS groups, the EPP group showed a significant increase in VH/CD and a significant decrease in CD in the duodenum (*p <* 0.001). Moreover, compared with the CON and LMS groups, the EPP group showed a significant increase in VH and VH/CD and a significant decrease in CD in the jejunum and ileum (*p <* 0.001). The results showed notable morphological differences in the small intestinal villi of broiler chickens among the EPP-supplemented, LMS, and CON groups. In the EPP-treated group, the intestinal villi were longer and more uniformly arranged. This structural improvement creates favourable conditions for enhanced nutrient absorption.

### 3.7 Intestinal antioxidant capacity

**Figure 5:**
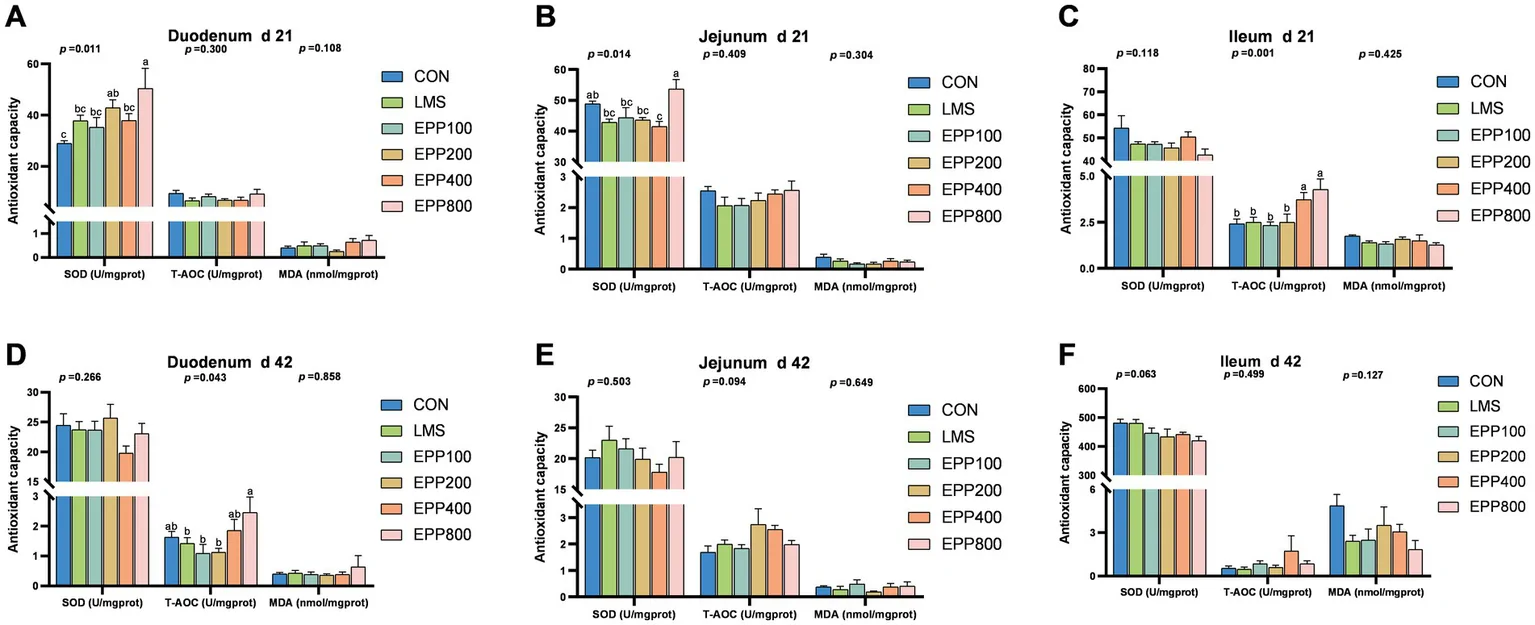
Effects of dietary EPP on intestinal antioxidant capacity in Mahuang chickens. **(A–F)** The antioxidant capacity indices (SOD, superoxide dismutase; T-AOC, total antioxidant capacity; and MDA, malondialdehyde) in the duodenum, jejunum, and ileum at days 21 **(A–C)** and 42 **(D–F)**. CON, fed with the basal diet; LMS, basal diet + Levamisole Hydrochloride 0.1 g/kg; EPP100, the basal diet + EPP 100 mg/kg; EPP200, the basal diet + EPP 200 mg/kg; EPP400, the basal diet + EPP 400 mg/kg; EPP800, the basal diet + EPP 800 mg/kg. Bars represent mean values ± SEM. The regressions were considered significant at *p*-value < 0.05. ^a,b,c^Means within the same row carrying different superscripts were significantly different at *p*-value < 0.05, while identical superscripts or no superscripts indicate no significant difference at *p*-value >0.05.

The impact of EPP on the activities of the intestinal antioxidant capacity of Mahuang chickens is shown in Figure 5. At 21 days of age, Duodenal SOD activity in the EPP200 and EPP800 groups was significantly higher than that in the CON group (*p =* 0.011) (Figure 5A). Jejunal SOD activity in the EPP800 group was significantly higher compared with the LMS groups (*p =* 0.014) (Figure 5B). Ileal T-AOC activity in the EPP400 and EPP800 groups was significantly higher than that of the CON and LMS groups (*p =* 0.001) (Figure 5C). At 42 days of age, Duodenal T-AOC activity in the EPP800 group was significantly higher compared with the LMS groups (*p =* 0.043) (Figure 5D). However, there were no significant differences in the SOD activity, T-AOC activity, and MDA content in the jejunum and ileum (*p >* 0.05) (Figures 5E,F). EPP is as effective as, or even more effective than, LMS in improving the local antioxidant status of the gut.

### 3.8 Intestinal immunoglobulin

**Figure 6:**
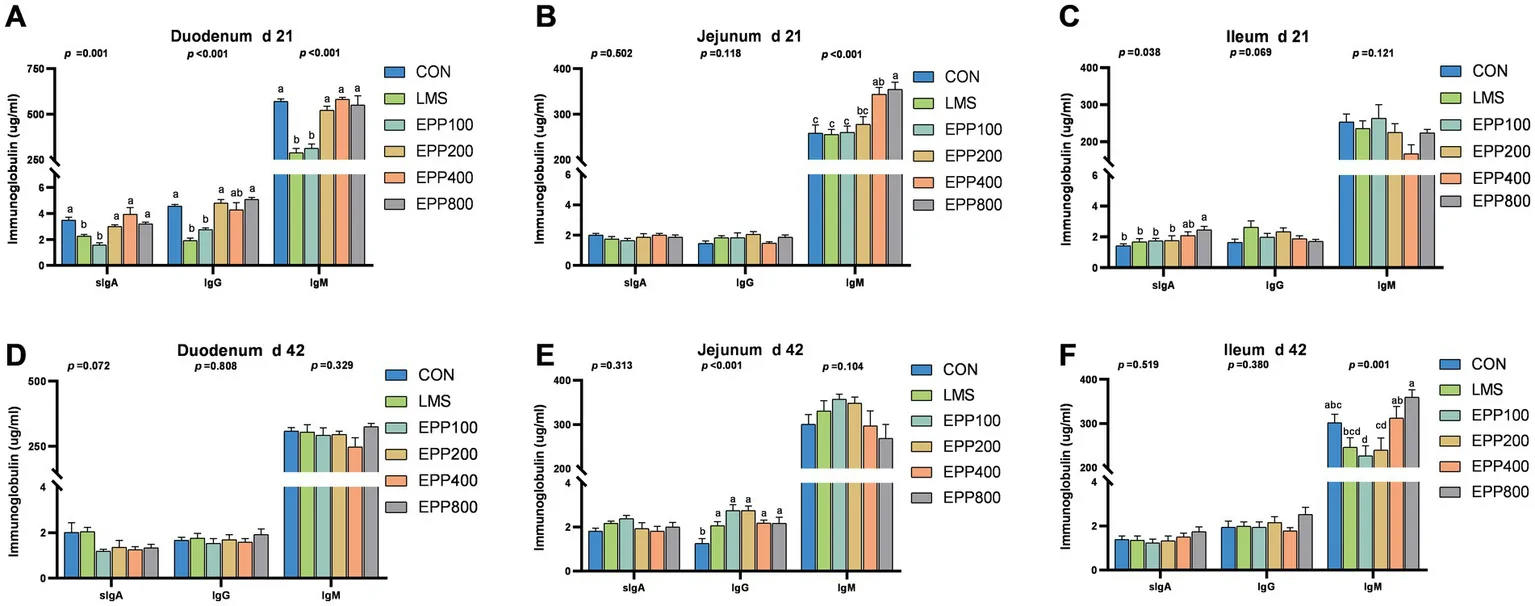
Effects of dietary EPP on immunoglobulin in Mahuang chickens. **(A–F)** The immunoglobulin indices (sIgA, Secretory Immunoglobulin A; IgG, Immunoglobulin G; IgM, Immunoglobulin M) in the duodenum, jejunum, and ileum at days 21 **(A–C)** and 42 **(D–F)**. CON, fed with the basal diet; LMS, basal diet + Levamisole Hydrochloride 0.1 g/kg; EPP100, the basal diet + EPP 100 mg/kg; EPP200, the basal diet + EPP 200 mg/kg; EPP400, the basal diet + EPP 400 mg/kg; EPP800, the basal diet + EPP 800 mg/kg. Bars represent mean values ± SEM. The regressions were considered significant at *p*-value < 0.05. ^a,b,c^Means within the same row carrying different superscripts were significantly different at *p*-value < 0.05, while identical superscripts or no superscripts indicate no significant difference at *p*-value >0.05.

The effects of dietary EPP on the levels of immunoglobulins in the duodenum, jejunum, and ileum of Mahuang chickens are shown in Figure 6. At 21 days of age, compared with the LMS group, the levels of sIgA (*p =* 0.001), IgG (*p <* 0.001), and IgM (*p <* 0.001) in the EPP200, EPP400, and EPP800 groups were significantly higher in the duodenum (Figure 6A). Compared with the CON and LMS groups, Jejunal IgM levels were significantly elevated in the EPP400 and EPP800 groups (*p <* 0.001) (Figure 6B). Ileal sIgA levels were significantly higher in the EPP800 group than those in the CON and LMS groups (*p =* 0.038) (Figure 6C). At 42 days of age, compared with the CON group, jejunal IgG levels were significantly higher in the treatment group (*p <* 0.001) (Figure 6E). In the ileum, the levels of IgM in the EPP800 group were significantly higher than those in the LMS group (*p =* 0.001) (Figure 6F). These results indicate that the addition of EPP to the diet is significantly more effective than, or at least equivalent to, LMS in promoting intestinal immunoglobulins, suggesting that it has good potential for use as a natural feed additive.

### 3.9 Gene expression

**Figure 7:**
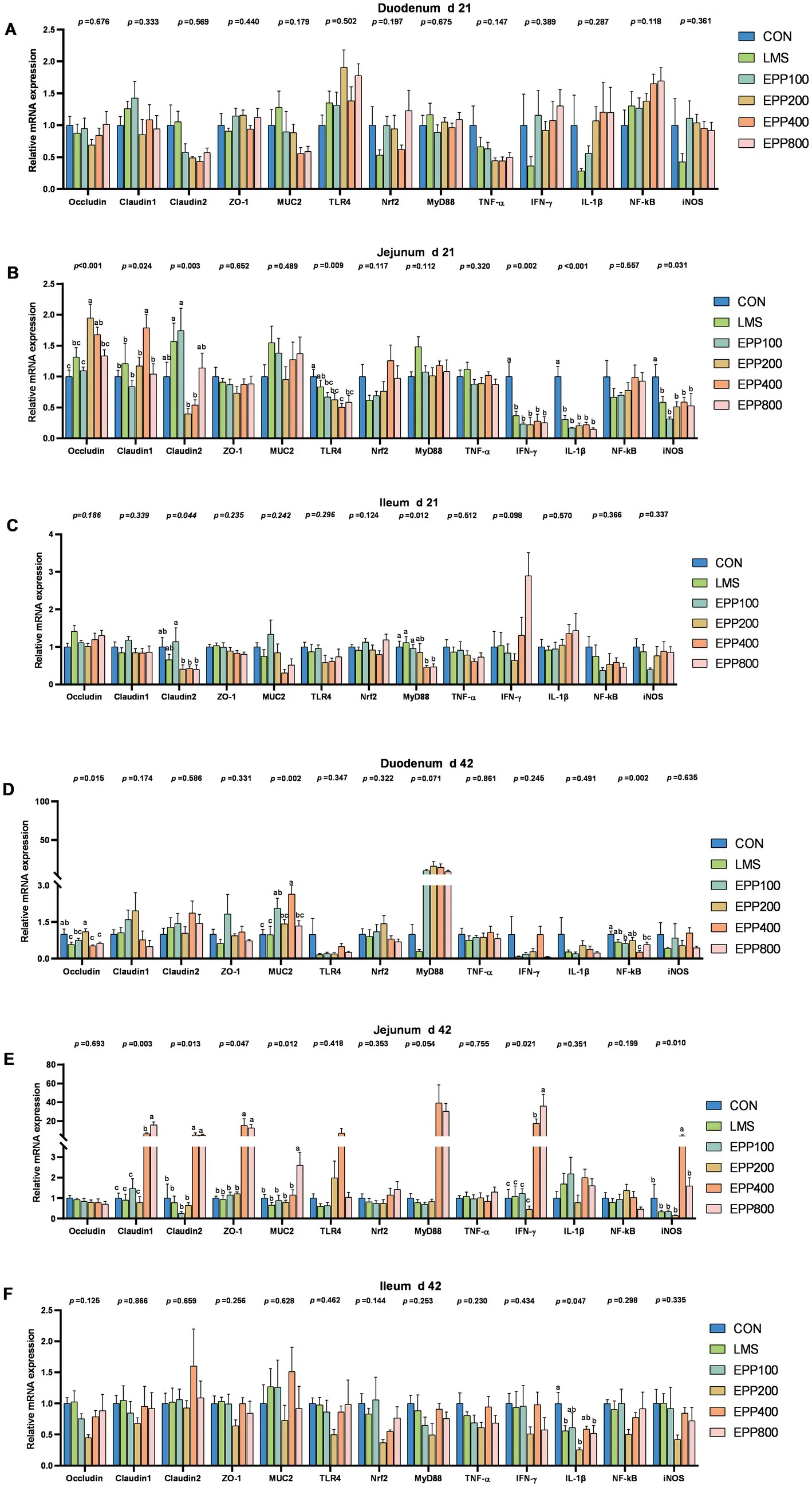
Effects of dietary EPP on the barrier- and immune-related gene expression in small intestinal of Mahuang chickens. **(A–F)** The barrier-related mRNA expression levels (Occludin; Claudin1; Claudin2; ZO-1, zonula occludens-1; MUC 2, mucin 2) and immune-related mRNA expression levels (TLR4, Toll-like receptor 4; MyD88, myeloid differentiation factor 88; TNF-α, tumor necrosis factor-α; IFN-γ, Interferon-gamma; IL-1β, Interleukin-1 beta; Nrf2, Nuclear factor erythroid 2-related factor 2; NF-κB, nuclear factor-kappa B; iNOS, Inducible nitric oxide synthase) in the duodenum, jejunum, and ileum at days 21 **(A–C)** and 42 **(D–F)**. CON, fed with the basal diet; LMS, basal diet + Levamisole Hydrochloride 0.1 g/kg; EPP100, the basal diet + EPP 100 mg/kg; EPP200, the basal diet + EPP 200 mg/kg; EPP400, the basal diet + EPP 400 mg/kg; EPP800, the basal diet + EPP 800 mg/kg. Bars represent mean values ± SEM. The regressions were considered significant at *P*-value < 0.05. ^a,b,c^Means within the same row carrying different superscripts were significantly different at *P*-value < 0.05, while identical superscripts or no superscripts indicate no significant difference at *P*-value >0.05.

Figure 7 illustrates the impact of dietary EPP on the relative intestinal barrier and immune gene expression in the duodenum, jejunum, and ileum of Mahuang chickens. On day 21, in the jejunum, the Occludin expression in the EPP100 and EPP400 groups (*p <* 0.001), and Claudin1 expression in the EPP400 group (*p =* 0.024) were increased significantly compared to the CON group. Conversely, the mRNA expression of TLR4 in the EPP groups (*p =* 0.009) was significantly decreased compared to the CON group. Moreover, the IFN-*γ* (*p =* 0.002), IL-1β (*p <* 0.001), and iNOS (*p =* 0.031) expression of the treatment groups were also down-regulated compared with the CON group (Figure 7B). In the ileum, the EPP400 and EPP800 groups had significantly decreased MyD88 mRNA expression compared to the CON and LMS groups (*p =* 0.012).

By day 42, the mRNA expression of MUC2 in the EPP100 and EPP400 groups (*p =* 0.002) was significantly increased compared with the CON group in the duodenum. In contrast, the NF-κB expression in the EPP100, EPP400, and EPP800 groups (*p =* 0.002) was decreased significantly compared to the CON group (Figure 7D). In the jejunum, the EPP400 and EPP800 groups had significantly higher Claudin1 (*p =* 0.003), Claudin2 (*p =* 0.013), ZO-1 (*p =* 0.047), and IFN-γ (*p =* 0.021) expression than the other group. Additionally, MUC2 in the EPP800 group (*p =* 0.012), and iNOS in the EPP400 group (*p =* 0.010) also exhibited differential increases compared with other groups (Figure 7E). In the ileum, the mRNA expression of IL-1β in the LMS, EPP200, and EPP800 groups (*p =* 0.047) was significantly lower than that in the CON group (Figure 7F). Overall, compared with LMS, EPP exerts a broader range of regulation and a more comprehensive effect on intestinal barrier homeostasis and the immune-inflammatory balance, demonstrating that EPP has considerable potential as a natural feed additive.

### 3.10 Gut microbiota structure and abundance

**Figure 8:**
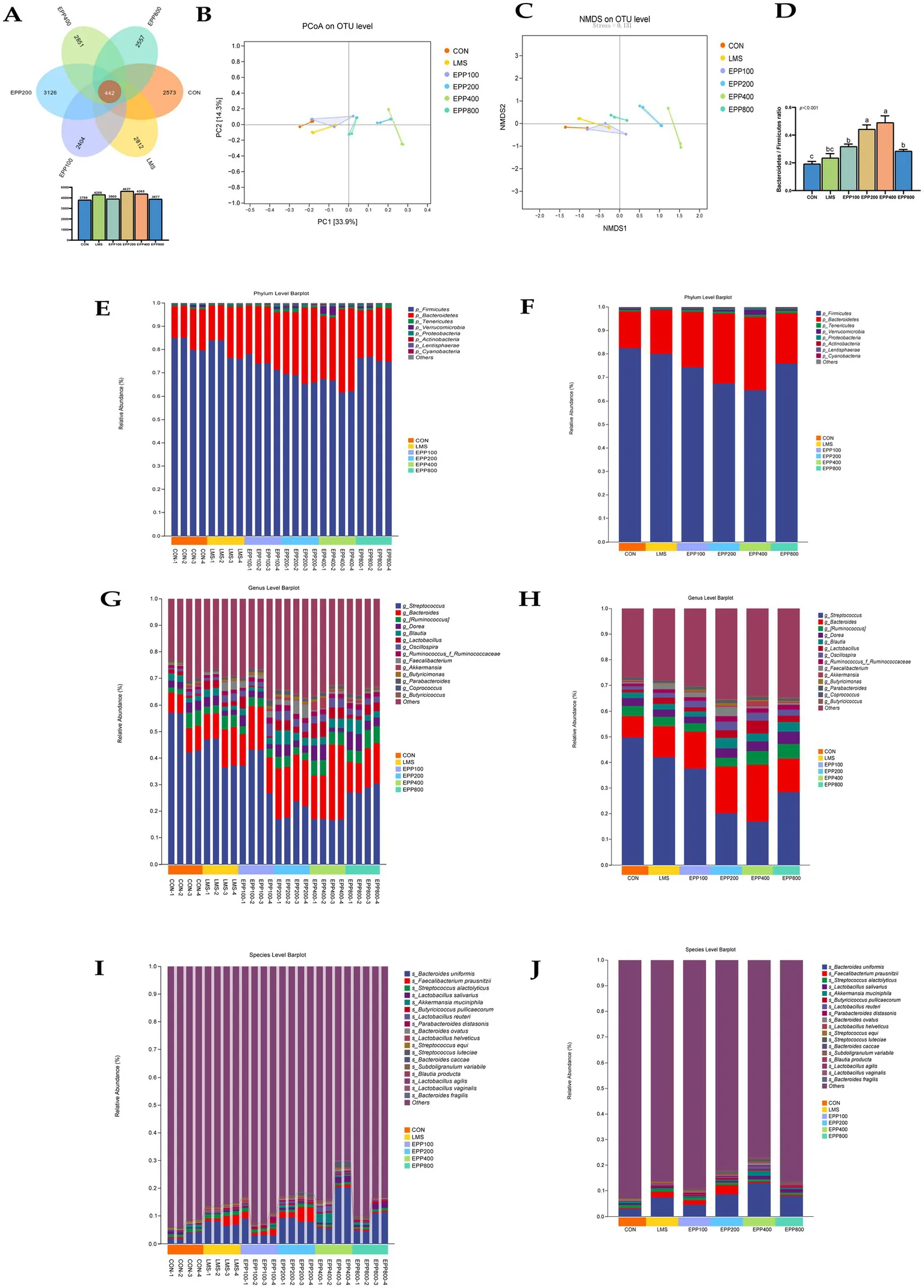
Effects of dietary EPP on the in intestinal microbiota of Mahuang chickens at 42 days of age. **(A)** petal Venn diagram. **(B)** Principal coordinate analysis (PCoA). **(C)** Nonmetric multidimensional scaling (NMDS) analysis. **(D)** Ratio of the phylum Bacteroidetes in Firmicutes. Microorganism community structures of the microbiota at phylum **(E,F)**, genus **(G,H)**, and species **(I,J)** levels. CON, fed with the basal diet; LMS, basal diet + Levamisole Hydro-chloride 0.1 g/kg; EPP100, the basal diet + EPP 100 mg/kg; EPP200, the basal diet + EPP 200 mg/kg; EPP400, the basal diet + EPP 400 mg/kg. EPP800, the basal diet + EPP 800 mg/kg.

The gut microbiota in different broiler groups was detected by 16S rRNA sequencing of the cecal content samples from 42-day-old broilers (Figure 8). After conducting sequencing and preliminary data assessment, each sample produced an average of 97,264 raw sequencing reads. Following quality trimming (to eliminate low-quality segments) and chimera filtering, an average of 63,788 high-quality sequences were retained per sample. In the present study, a petal Venn diagram was employed to illustrate the average number of operational taxonomic units (OTUs) distribution characteristics of cecal microbiota in six groups (CON, LMS, EPP100, EPP200, EPP400, and EPP800) of broilers. Each petal area represents group-specific OTUs of the corresponding group, while the central overlapping area represents the core OTUs shared by all groups. Results from the petal Venn diagram revealed that the number of group-specific OTUs was 2,573, 2,812, 2,401, 3,126, 2,851, and 2,557 for the CON, LMS, EPP100, EPP200, and EPP400 groups, respectively, while 442 OTUs were shared among all groups (Figure 8A). Regarding community similarity, bray–curtis distance-based principal coordinate analysis (PCoA) and nonmetric multidimensional scaling (NMDS) of the cecal microbial community were performed to evaluate the similarities and dissimilarities across the six groups. The results demonstrated that, in comparison with the CON group, the microbial compositions of the LMS, EPP200, EPP400, and EPP800 groups formed distinctly separated clusters (Figures 8B,C).

At the phylum level, the most abundant phyla of intestinal microbiota across all experimental groups were Firmicutes and Bacteroidetes. Compared to the CON and LMS groups, the abundance of Bacteroidetes in the EPP group was consistently increased. The B/F ratio in the EPP200 and EPP400 groups was significantly higher than that in the other groups, and in the EPP100 and EPP800 groups was significantly higher than the CON group (*p* < 0.001). Furthermore, the abundance of Verrucomicrobia in the EPP400 group was higher than that in all other groups (Figures 8D–F).

At the genus level, the abundance of *g_Streptococcus* bacteria in the CON, LMS, and EPP100 groups was higher than that in the EPP200, EPP400, and EPP800 groups (*p* < 0.001), while *g_Blautia* was observed to be reduced compared to that in the EPP200 group (*p* = 0.031). Compared with the EPP400 group, the abundance of *g_Bacteroides* (*p* < 0.001), *g_Lactobacillus* (*p* = 0.003), and *Akkermansia* (*p* = 0.042) was reduced in all other groups. Compared with the CON and LMS groups, *g_Oscillospira* (*p* = 0.022) of the EPP400 group, *g_Ruminococcus_f_Ruminococcaceae* of the EPP200, EPP400, and EPP800 groups, and *g_Butyricicoccus* (*p* = 0.009) of the EPP groups had a greater relative abundance (Figures 8G, 5H).

At the species level, the abundance of *s_Bacteroides uniformis* (*p* = 0.038) in the EPP400 group and *s_Faecalibacterium prausnitzii* (*p* = 0.022) in the EPP200 group was higher than that in the CON group. The relative abundance of *s_Lactobacillus salivarius* (*p* < 0.001) in the EPP400 and EPP800 groups, and *s_Blautia producta* (*p* = 0.017) in the EPP100 and EPP400 groups, was significantly greater than that in the LMS group. Additionally, compared to the CON and LMS groups, the EPP200 and EPP800 groups exhibited an enhanced proportion of *s_Butyricicoccus pullicaecorum* (*p* = 0.009), the EPP100, EPP200, and EPP800 groups showed an increased proportion of *s_Lactobacillus agilis* (*p* < 0.001), and the EPP200 group had a higher proportion of *s_Lactobacillus vaginalis* (*p* = 0.007), whereas the proportion of *s_StrePtococcus equi* (*p* < 0.001) was reduced in all EPP groups. Moreover, broilers fed the EPP400 diet exhibited increased relative abundances of *s_Akkermansia muciniphila* (*p* = 0.040), *s_Lactobacillus reuteri* (*p* = 0.012), and *s_Bacteroides ovatus* (*p* < 0.001) compared to all other groups (Figures 8I,J).

### 3.11 Correlation analysis between microbe and different indicators

**Figure 9:**
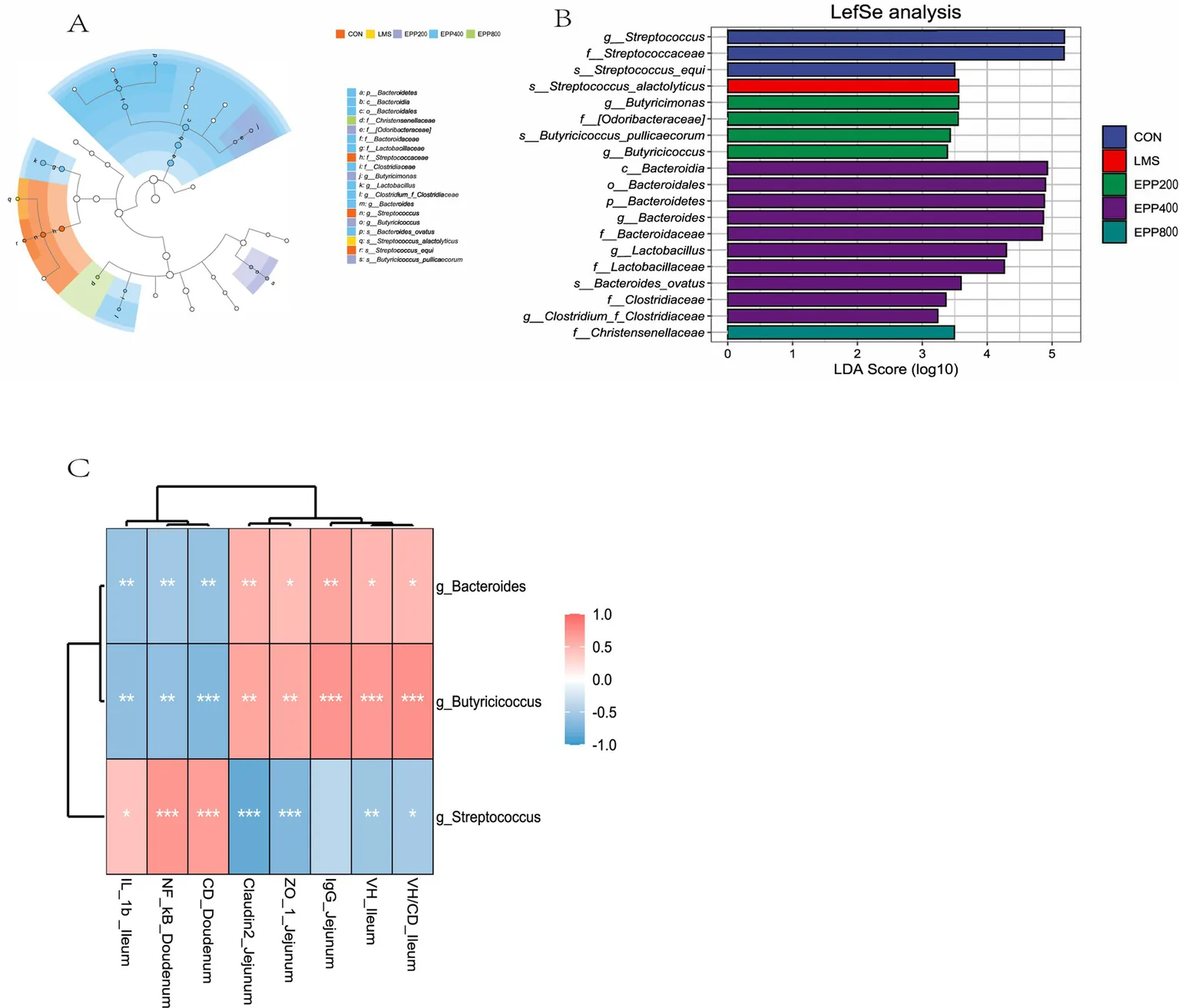
Correlation analysis between the intestinal microbiota and different indicators. **(A,B)** Linear discriminant analysis (LDA) Effect Size (LEfSe) analysis (LDA > 3.0, *P* < 0.05). **(C)** Spearman’s correlation analysis of the LEfSe analysis and differential indicators. CON, fed with the basal diet; LMS, basal diet + Levamisole Hydro-chloride 0.1 g/kg; EPP100, the basal diet + EPP 100 mg/kg; EPP200, the basal diet + EPP 200 mg/kg; EPP400, the basal diet + EPP 400 mg/kg. EPP800, the basal diet + EPP 800 mg/kg. *indicates a significant difference at *p* < 0.05, **indicates *p* < 0.01, ***indicates *p* <0.001.

Figure 9A presents the results of the Linear discriminant analysis Effect Size (LEfSe) method, revealing 3, 1, 4, 10, and 1 microbial taxa with significantly different abundances in the CON, LMS, EPP200, EPP400, and EPP800 groups, respectively (LDA score > 3.0). The results indicated that *g_Streptococcus* (LDA = 5.223, *p* = 0.001), f_Streptococcaceae (LDA = 5.186, *p* = 0.002), and *s_Streptococcaceae* equi (LDA = 3.498, *p* = 0.002) were significantly enriched in the CON group, with *g_Streptococcus* and f_Streptococcaceae exhibiting the highest LDA scores (approximately 5.0). The dominant cecal microflora enriched in the LMS group was *s_Streptococcus alactolyticus* (LDA = 3.559, *p* = 0.005). The EPP200 group showed significant enrichment of bacteria, including *g_Butyricimonas* (LDA = 3.558, *p* = 0.022), f_Odoribacteraceae (LDA = 3.554, *p* = 0.022), *s_Butyricicoccus_pullicaecorum* (LDA = 3.430, *p* = 0.010), and *g_Butyricicoccus* (LDA = 3.387, *p* = 0.010). In contrast, the EPP400 group was enriched with a broad range of beneficial microbial taxa across multiple taxonomic levels: c_Bacteroidia (LDA = 4.929, *p* = 0.001), *o*_Bacteroidales (LDA = 4.899, *p* = 0.001), *p_ Bacteroidetes* (LDA = 4.882, *p* = 0.001), *g_Bacteroides* (LDA = 4.867, *p* = 0.002), f_Bacteroidaceae (LDA = 4.850, *p* = 0.002), *g_Lactobacillus* (LDA = 4.296, *p* = 0.014), f_Lactobacillaceae (LDA = 4.263, *p* = 0.014), *s_Bacteroides_ovatus* (LDA = 3.184, *p* = 0.022)*,* f_Clostridiaceae (LDA = 3.364, *p* = 0.022), and *g_Clostridium_f_Clostridiaceae* (LDA = 3.184, *p* = 0.022). The family significantly enriched in the EPP800 group included *f_Christensenellaceae* (LDA = 3.505, *p* = 0.024).

Figure 9B presents the conceivable interconnections between the main cecal microbiota genus and all significantly different parameters that were examined in this study using a Spearman correlation analysis. In this study, Bacteroides and Butyricicoccus demonstrated positive correlations with IgG in the jejunum (*p* < 0.01). The abundance of Bacteroides and Butyricicoccus showed positive correlations with VH/CD (Ileum), VH (Ileum), ZO-1expression (Jejunum), and Claudin2 expression (Jejunum) (*p* < 0.05) and negative correlations with the CD (Duodenum) and the mRNA expression of NF-kB (Duodenum) and IL-1β (Ileum) (*p* < 0.01). Whereas the abundance of Streptococcus was positively correlated with the CD (Duodenum) and the mRNA expression of NF-kB (Duodenum) and IL-1β (Ileum) (*p* < 0.05) and negatively correlated with VH/CD (Ileum), VH (Ileum), ZO-1expression (Jejunum), and Claudin2 expression (Jejunum) (*p* < 0.05), respectively.

### 3.12 Functional prediction of cecal microbes

**Figure 10:**
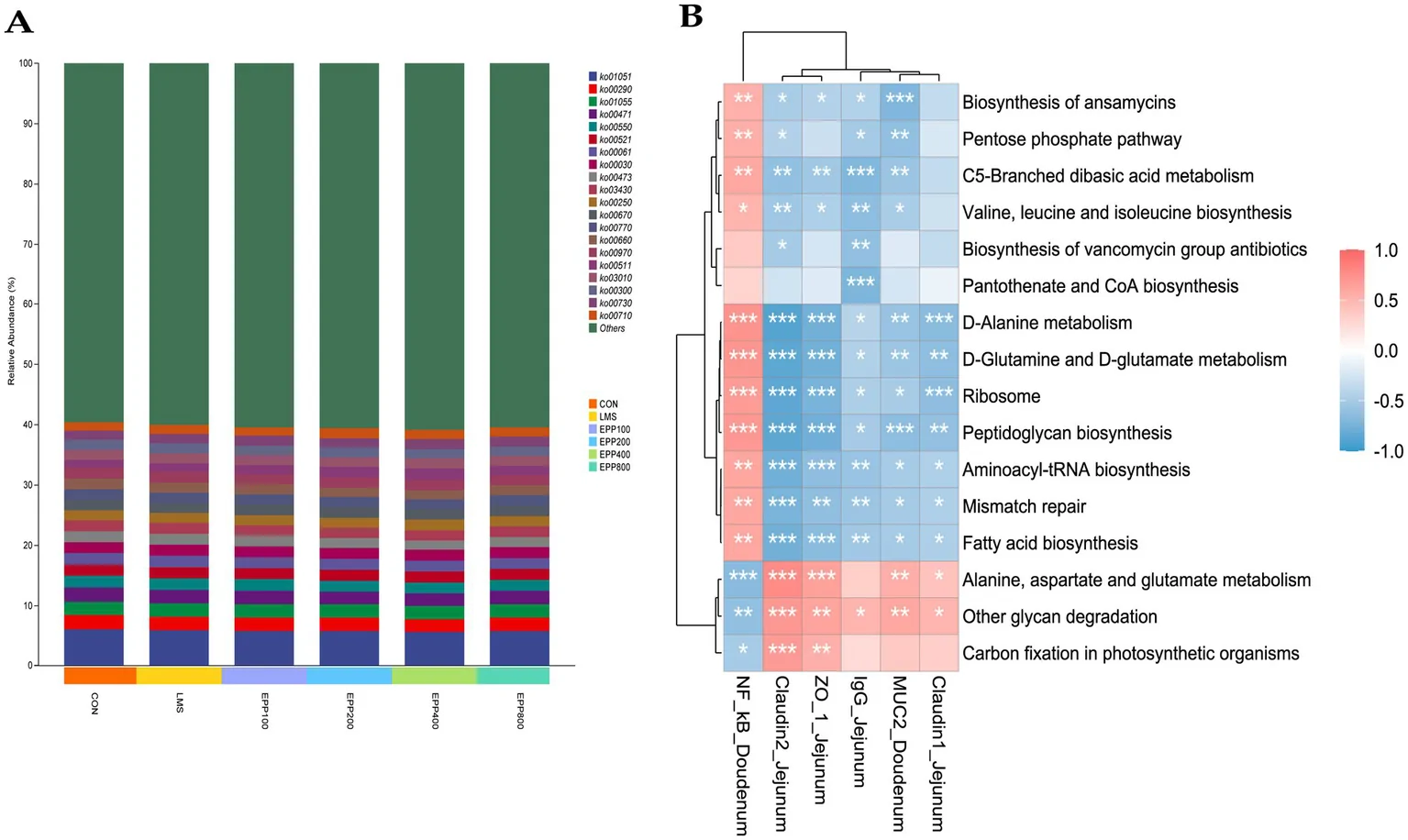
Correlation analysis between the predicted function and different indicators. **(A)** PICRUSt2 KEGG. **(B)** Spearman’s correlation analysis of the PICRUSt2 KEGG and differential indicators. CON, fed with the basal diet; LMS, basal diet + Levamisole Hydro-chloride 0.1 g/kg; EPP100, the basal diet + EPP 100 mg/kg; EPP200, the basal diet + EPP 200 mg/kg; EPP400, the basal diet + EPP 400 mg/kg. EPP800, the basal diet + EPP 800 mg/kg. *indicates a significant difference at *p* < 0.05, **indicates *p* < 0.01, ***indicates *p* <0.001.

The changes in the metabolic pathways of the predictive functional profiles from the cecal microbiota and all significantly different parameters are shown in Figure 10. The metabolic pathways of glycan degradation, alanine, aspartate, glutamate metabolism, and carbon fixation in photosynthetic organisms show positive correlations with Intestinal tight junctions and intestinal morphology. Conversely, the negative correlations with the NF-kB (Duodenum) and the CD (Duodenum). Furthermore, the upper half of the figure shows that pathways including the biosynthesis of ansamycins, the pentose phosphate pathway, C5-branched dicarboxylic acid metabolism, and the biosynthesis of vancomycin-class antibiotics are significantly positively correlated with pro-inflammatory factors (NF-κB, Duodenum) and CD (Duodenum) (*p* < 0.05). Conversely, these pathways showed a significant negative correlation with tight junction proteins (Claudin 2, ZO-1), the VH/CD, and IgG levels. As shown in the centre of the figure, pathways such as D-alanine metabolism, peptidoglycan biosynthesis, and ribosomes exhibit strong bidirectional correlations. These pathways showed a highly significant positive correlation (*p* < 0.05) with the inflammatory factor NF-κB and CD, but a significant negative correlation with the junctional proteins Claudin2 and ZO-1, as well as with the morphological indicator VH/CD (*p* < 0.05). This suggests that the enrichment of these metabolic functions may be associated with an exacerbated inflammatory response in the gut and impaired barrier function.

## 4 Discussion

Numerous studies have shown that phytogenic feed additives and extracts, especially plant polysaccharides, have become increasingly popular in enhancing broiler health, owing to their multiple functions in promoting growth, antioxidation, immunoregulation, and intestinal barrier enhancement (24, 25). In our study, Mahuang chickens fed diets supplemented with different doses of EPP revealed that, between 1 and 21 days of age, BWG was significantly increased in the EPP100, EPP200, EPP800, and LMS groups. Notably, EPP did not sustain significant BWG differences from 28 to 42 days. These results indicated that EPP exerted positive effects on the early growth performance of Mahuang broilers. This is most likely attributable to the fact that EPP improves the digestion, absorption, and utilization of feed by regulating the gut microbiota, thereby leading to optimized body weight. Previous studies have shown that days 1–21 of a chick’s life represent a critical window for the rapid assembly of the caecal microbiota and the establishment of its homeostasis (26). Consequently, the immature gut microbiota is more sensitive to exogenous plant polysaccharide intervention. However, after 21 days of age, the microbial flora in the caecum of Mahuang broilers gradually matures and reaches a stable symbiotic equilibrium, resulting in reduced sensitivity to regulation by exogenous EPP (27). This leads to a diminished growth-promoting effect of polysaccharides; consequently, no significant differences in weight gain were observed during the later stages.

Organ index also reflects poultry growth, metabolism, and immunity to a certain extent. Previous studies have shown that 500 mg/kg Chinese yam polysaccharides enhanced thymus and spleen indices in broilers (28), improving macrophage phagocytic function and host immunity (29). Cai et al. (30) found that 1.2 g/kg supplementation of Alhagi honey polysaccharides significantly increased the index of the thymus, spleen, and bursa of fabricius in Hy-Line Brown chickens. Different from previous research, this study revealed that dietary supplementation with EPP had no significant effect on the organ indices of Mahuang chickens. This discrepancy may be attributed to differences in polysaccharide content, source, and broiler breeds (e.g., Hy-Line Brown chicken, Arbor Acres broiler, yellow-feathered broiler, pheasant, and Mahuang chickens). These results also indicate that EPP is safe for broiler organ development within the tested dosage and experimental period. Moreover, Li et al. (31) demonstrated that combined dietary supplementation with Astragalus polysaccharides and probiotics (Lactobacillus and *Bacillus cereus*) exerted a synergistic regulatory effect on immune function and intestinal microbiota, with significant differences in spleen index, BF index, and immune organs histopathology compared with single Astragalus polysaccharides or probiotic supplementation. Therefore, exploring the combined probiotics and EPP supplementation may have a more pronounced effect on organ indices than single polysaccharide supplementation, which warrants further investigation.

Serum biochemical indicators serve as critical indicators of the animal’s health status, protein synthesis capacity, metabolic energy capacity, and immune function (32). More specifically, urea is commonly used to assess renal function in animals, whilst ALT, AST, and ALP are markers of liver function (33). TC, TG, ALB, TP, and GLB are commonly used to assess the body’s ability to absorb and metabolize lipids and proteins (34, 35). Our findings revealed that different doses of EPP had no significant effect on the serum biochemical indicators of Mahuang broilers from day 1 to 42, consistent with the findings of other studies (36). This suggests that EPP did not cause hepatotoxicity, nephrotoxicity, or metabolic disorders in MaHuang chickens, demonstrating its good overall safety profile. This finding is particularly noteworthy, as previous studies have reported that certain synthetic feed additives or synthetic alternatives to antibiotics can have adverse effects on liver and kidney function in animals when used at high doses or over prolonged periods (37). Furthermore, the maintenance of serum lipid and protein homeostasis suggests that EPP does not interfere with normal nutrient absorption, transport, or utilization pathways. Taken together with the consistent growth performance and organ index data, these results highlight the potential of EPP as a safe, natural feed additive.

Antioxidant enzymes are recognised for their significant role in contributing to antioxidant defense mechanisms (38). Studies have suggested that plant polysaccharides exert antioxidant effects indirectly by enhancing antioxidant enzyme activities (39, 40). In the current study, 800 mg/kg EPP significantly increased duodenal SOD activity, while 400 and 800 mg/kg EPP elevated ileal T-AOC activity. In particular, at 21 days of age, it demonstrated effects superior to or equivalent to those of the positive control LMS, enhancing the enzymatic antioxidant defence system at the intestinal level (22, 41). This suggests that the weight gain in the MaHuang chickens at 21 days of age may be due to increased antioxidant levels in the gut during the early stages, which improved gut health and consequently enhanced BWG. The gut is the primary site of oxidative stress in poultry induced by feed components, pathogens, and environmental stressors (42). By enhancing the gut’s antioxidant defences, EPP helps to maintain the integrity of the intestinal epithelium and reduce oxidative damage, thereby supporting nutrient absorption and overall gut health, but the exact underlying mechanism requires further investigation. A comparison with the results from LMS suggests that EPP shows promise as a natural feed additive that promotes poultry gut health in a safer and more sustainable manner.

Currently, numerous plant polysaccharides are used to maintain the health of chickens through improving the intestinal function and immunity, and the immunoglobulins are the main components of humoral immunity (43, 44). Poultry possess three types of immunoglobulins (IgG, IgA, and IgM), which exhibit functions such as antiviral, antibacterial, and immunomodulatory activities (45, 46). Taking into account the combined results from the serum and intestinal, although EPP may cause transient fluctuations in serum sIgA levels in the early stages, it does not have any adverse effects on the body. On the contrary, it may promote the transport of immune effector molecules to the gut (47). In this study, broiler chickens fed diets supplemented with 400 and 800 mg/kg of EPP showed a significant increase in intestinal sIgA, IgG, and IgM levels, with the effects generally being superior to or equivalent to those observed in the LMS. The intestine is the largest immune organ in poultry, and EPP significantly enhances the intestinal immune function of Ma Huang chickens, which is of great importance for resisting orally transmitted pathogens and ensuring healthy intestinal development.

The intestine is an important organ for digestion, absorption, and immune defense in animals. VH and CD are key indicators reflecting intestinal health and digestive-metabolic status in chickens (48). This study found that in the EPP treatment group, the intestinal villi were well-extended and neatly arranged. Additionally, adding EPP to diets enhanced VH and VH/CD, whilst CD was significantly reduced in the duodenum, jejunum, and ileum of MaHuang chickens. These morphological improvements indicate that EPP promotes a healthier and more functionally mature intestinal epithelium, as evidenced by the elongation of villi to increase the surface area for nutrient transport, the shallowing of crypts to reduce the energy expenditure associated with cell proliferation, and a higher VH/CD, which enhances the small intestine’s capacity for nutrient absorption (49, 50). This structural optimisation is particularly beneficial during the early growth stages, when rapid weight gain requires maximum nutrient assimilation. Importantly, the EPP treatment group consistently demonstrated results that were superior to or equivalent to those of the positive control, LMS, in terms of intestinal morphology. The underlying mechanisms may involve several pathways: firstly, an improvement in the gut’s antioxidant capacity may protect intestinal cells from oxidative stress-induced apoptosis, thereby maintaining villous integrity (51). Secondly, elevated levels of immunoglobulins in the gut may help to create a healthier intestinal environment by controlling the pathogen load and reducing inflammatory damage to the villi (52, 53). Thirdly, EPP may promote the growth of intestinal villi by improving the composition of the gut microbiota (1, 54). These interrelated mechanisms collectively account for the significant morphological improvements observed in chickens fed EPP, although their precise mechanisms of action require further investigation. In addition, it may also be beneficial for intestinal barrier function. The intestinal barrier function maintains the stability of the intestinal environment and prevents the invasion of harmful substances, which is primarily maintained by Occludin, Claudins, ZO-1, and MUC2 to ensure intestinal barrier integrity (55). The results of this study show that, at 21 days of age, the mRNA expression levels of both Occludin and Claudin 1 in the jejunum of the EPP400 group were significantly upregulated. At 42 days of age, the gene expression levels of Claudin1, Claudin2, and ZO-1 in the jejunum of the EPP400 and EPP800 groups were significantly elevated. These findings indicate that EPP is capable of maintaining the integrity of the tissue structure, promoting intestinal development and barrier integrity (56, 57), aiding in the clearance of intestinal pathogens (31), and regulating intestinal immunity (58), thereby maintaining normal intestinal physiological function (59, 60). In this study, at 42 days of age, MUC2 mRNA expression levels were significantly elevated in the duodenum of the EPP100 and EPP400 groups and in the jejunum of the EPP800 group. This indicates that EPP plays a key role in protecting the mucous membranes from damage and in resisting the invasion of pathogens (61, 62). The synergistic enhancement of both tight junction proteins and mucins—components of the structural and secretory barriers—suggests that EPP promotes epithelial cell differentiation and barrier maturation. However, the specific mechanisms underlying EPP-mediated immunoregulation require further investigation. Previous studies have shown that inflammatory stress downregulates the gene expression of claudin-1, occludin, and ZO-1 in the intestines of broiler chickens and piglets, thereby increasing permeability between intestinal epithelial cells (63, 64). This allows harmful substances to penetrate epithelial cells and release large amounts of inflammatory cytokines (65, 66), whilst the mRNA expression of TLR4, MyD88, NF-κB, IL-1β and TNF-*α* in the intestine increases significantly (67). In contrast, this experiment did not reveal any significant up-regulation of these immune-related genes. We observed a significant down-regulation of TLR4 and MyD88 expression, whilst the expression of downstream inflammation-related mediators such as IL-1B, IFN-*γ* and iNOS was also reduced. In addition to enhancing the barrier effect, EPP continuously downregulates key components of the TLR4/MyD88/NF-κB signalling pathway, thereby creating a more favourable environment for the absorption of nutrients and tissue growth. At 42 days of age, the EPP group showed a significant upregulation of tight junction proteins and mucins, accompanied by a moderate increase in IFN-γ and iNOS. Additionally, IFN-γ, as a macrophage-activating factor (68, 69), can promote the protective effects of macrophages by regulating the expression of inducible nitric oxide synthase (iNOS) (70–72). This indicates that, during the late developmental stage of broiler chickens, EPP continuously and effectively stabilises the intestinal barrier whilst maintaining an appropriate physiological immune response in the host, rather than continuously and unilaterally suppressing inflammation. Taken together, these findings suggest that EPP activates macrophages and stimulates the host immune system by regulating the expression of IFN-γ and iNOS, without compromising the host’s defences. However, the underlying mechanisms require further investigation.

The intestinal microbial community in the animal intestine plays a crucial role in preserving gut homeostasis, supporting physiological functions, and regulating the host immune system (73). This study used 16S rRNA sequencing to analyze the regulatory effects of different doses of EPP interventions on the cecal microbiota of broilers. Both principal coordinate analysis (PCoA) and non-metric multidimensional scaling (NMDS) revealed that the microbial communities in the EPP200, EPP400, and EPP800 groups formed distinct clusters that were clearly separated from the CON group, whereas those in the LMS group showed greater similarity to the CON group. These findings indicate that EPP intervention significantly altered the overall structure of the cecal microbiota. Our study found that dietary supplementation with EPP increased the B/F ratio, which is consistent with the findings reported by Tang et al. (74). This indicates that the addition of EPP can improve the integrity of the mucus layer and intestinal barrier function by regulating the composition of the gut microbiota (75). These findings are consistent with the results regarding intestinal morphology and immune-related parameters presented earlier and further elucidate the underlying mechanism by which EPP maintains normal intestinal physiological function in Mahuang-fed chickens. The results of this study show that, at the genus level, EPP supplementation increased the abundance of *Bacteroides*, *Blautia*, *Lactobacillus*, *Oscillospira*, *Ruminococcus* (family *Ruminococcaceae*), *Akkermansia*, and *Butyricicoccus*. These genera are widely recognized as major producers of SCFAs, including butyrate, acetate, lactate, and succinate, which are essential for maintaining energy supply to the intestinal epithelial cells and an anti-inflammatory environment (76–81). In contrast, the relative abundance of harmful bacteria (*Streptococcus*) increased in the CON and LMS-supplemented groups. These findings indicate that EPP can enhance the relative abundance of beneficial bacteria and suppress the growth of potentially harmful bacteria, with its efficacy superior to that of the reference drug (levamisole). At the species level, EPP showed a significant enrichment of several key species within the genus Bacteroides, including *B. uniformis* and *B. ovatus*. These microorganisms can degrade and metabolise dietary polysaccharides, providing nutrients for other gut symbiotic bacteria (82, 83). At the same time, they perform immune and defence functions, working synergistically to strengthen the stability of the intestinal barrier (84, 85). Regarding probiotics, EPP significantly promoted the proliferation of various *Lactobacillus strains*, such as *L. salivarius*, *L. agilis*, *L. reuteri*, and *L. vaginalis*. *Lactobacillus* species possess functions including acid production, bacteriostasis, immune regulation, and improvement of the intestinal environment (86–89). This increase in abundance lays the foundation for the stability of the gut microbiota. In particular, a significant increase in the abundance of *L. salivarius* markedly improves the B/F ratio and promotes villus growth in all intestinal segments (54), which is consistent with the findings of this study. Furthermore, both the CON group and the LMS-supplemented group exhibited an increase in the abundance of *Streptococcus equi*. Conversely, EPP significantly inhibited the proliferation of *Streptococcus equi*. As a potential pathogen, a reduction in its abundance helps to lower the risk of infection and alleviate inflammatory stress (90, 91). Furthermore, the increase in the abundance of *Blautia producta* in the EPP-treated group further enhanced intestinal fermentation and barrier function (92). At the same time, EPP significantly increased the abundance of butyrate-producing bacteria, particularly *F. prausnitzii* and *Butyricicoccus pullicaecorum* (93, 94). Butyric acid promotes the expression of tight junction proteins, thereby improving barrier integrity (108). This explains why 400 mg/kg and 800 mg/kg of EPP significantly increased the relative expression levels of gut barrier-related genes in MaHuang chickens and enhanced barrier integrity. However, the underlying mechanisms require further investigation. Furthermore, the significant enrichment of *Akkermansia muciniphila* further corroborates the potential of EPP in repairing the intestinal mucosa, modulating metabolism, and exerting anti-inflammatory effects (95). Overall, EPP can modulate the structure of the intestinal microbiota through multiple dimensions. In particular, the 400 mg/kg EPP dosage effectively promotes a shift in the microbiota towards a healthier composition. Compared with the CON group and the positive control group (LMS), EPP400 demonstrated superior performance in promoting the proliferation of beneficial bacteria, inhibiting harmful bacteria, and increasing the abundance of key functional bacterial species. These findings suggest that EPP holds significant potential as a novel poultry feed additive for improving gut health.

Spearman’s correlation analysis revealed that Bacteroides and Butyricicoccus were positively correlated with gut integrity and immunoglobulins and strongly negatively correlated with all inflammatory markers. In this study, Bacteroides is a core gut microbiota that is involved in polysaccharide degradation and the production of SCFAs. As a butyrate-producing bacterium, Butyricicoccus helps to improve the intestinal barrier, boost immunity, and promote the development of small intestinal villi (78). Cluster analysis reveals that g_Bacteroides and g_Butyricicoccus act synergistically to exert protective effects by reducing inflammation, repairing the intestinal barrier, promoting villus development and enhancing mucosal immunity. This suggests that EPP may improve the normal development of intestinal villi and maintain gut homeostasis by regulating Bacteroides and Butyricicoccus. Overgrowth of the genus Streptococcus may be associated with impaired intestinal barrier function and inflammatory responses (91). Therefore, supplementing broiler feed with EPP not only enriches beneficial bacteria and inhibits harmful bacteria, but also helps to maintain intestinal integrity, regulate the immune system, and reduce inflammation.

This study conducted a Spearman correlation analysis based on PICRUSt2 functional prediction, providing clear evidence that KEGG metabolic pathways of the gut microbiota exhibit bidirectional antagonistic regulation of inflammation, the epithelial barrier, mucosal immunity and tissue morphology in the small intestine of poultry; hierarchical clustering categorised 15 key pathways into three modules with distinctly opposite functions. Firstly, functional clusters positively correlated with inflammation and barrier damage. These pathways showed a significant negative correlation with tight junction proteins, intestinal morphology, and IgG levels, suggesting that the accumulation of these metabolic functions may be associated with exacerbated intestinal inflammation and impaired barrier function (96). Secondly, the complex pathways associated with bacterial cell wall synthesis and basal metabolism showed a highly significant positive correlation with inflammatory factors and CD. But a significant negative correlation with tight junction proteins and morphological indicators in the jejunum (97). This suggests that the active metabolism of bacterial cell wall components may be closely associated with local immune activation and tissue remodelling. Thirdly, functional clusters positively correlated with barrier repair and immune homeostasis showed a significant positive correlation with intestinal barrier expression, whilst exhibiting a significant negative correlation with inflammatory cytokines (98). This suggests that EPP may maintain intestinal epithelial integrity and suppress inflammation by stimulating these metabolic activities. Consequently, supplementation with EPP may help to promote the growth of functional microbiota that degrade amino acids and carbohydrates, thereby restoring intestinal barrier function.

## 5 Conclusion

To sum up, Mahuang chickens fed EPP-supplemented diets displayed better growth performance and intestinal health than those in the CON and LMS groups, with superior effects observed in the high-dose groups (EPP400, EPP800) relative to the low-dose groups (EPP100, EPP200). These beneficial effects were characterized by improved antioxidant function, immunity, intestinal morphology, the expression of intestinal barrier- and immune-related genes, and microbial communities. Among them, 400–800 mg/kg EPP of EPP used in this study had the best effect for Mahuang chickens, but the potential mechanisms of action of EPP in Mahuang broilers remain to be further investigated.

## Data Availability

The 16S rRNA raw data supporting the conclusions of this article have been uploaded to the repository: https://ngdc.cncb.ac.cn/gsa/ (GSA: CRA048246).

## Footnotes

1
https://www.genescloud.cn
